# A Rare Case of Gastrointestinal Mucormycosis

**DOI:** 10.7759/cureus.17748

**Published:** 2021-09-05

**Authors:** Jasneet Kaur, Upinder Singh, Uma Pradhan, Gulshan Singh, Prem Narayan Agarwal

**Affiliations:** 1 Department of Pathology, Genomics Laboratory, New Delhi, IND; 2 Department of General Surgery, Shree Guru Gobind Singh Tricentenary University, Gurugram, IND

**Keywords:** gastrointestinal mucormycosis, ileocecal mucormycosis, typhoid infection, melaena, right hemicolectomy

## Abstract

Gastrointestinal Mucormycosis (GIM) is a rare life-threatening angio-invasive infection. The classic risk factors include immunosuppression and metabolic derangement. Usually, there are classical risk factors in patients affected by Ileocecal mucormycosis. Few case reports have shown the absence of salient clinical presentation of mucormycosis in prolonged hospitalisation. The presence of association of mucormycosis in patients of typhoid infection is rare. Here, we present a case of invasive ileal mucormycosis occurring as a sequel to typhoid infection which lacked the typical risk factors for mucormycosis.

## Introduction

Gastrointestinal (GI) mucormycosis is an uncommon, usually opportunistic, life-threatening angio-invasive infection, and accounts for 4 to 7 % of all cases of mucormycosis [1,2]. Traditional risk factors include diabetes, corticosteroid use, immunocompromised state, transplant patients on immunosuppressive therapies and iron overload states with or without deferoxamine therapy, and malnutrition [3]. The association of mucormycosis with typhoid infection is extremely rare [4]. We present here a case of invasive ileal mucormycosis occurring as a sequel to typhoid infection in absence of typical risk factors.

## Case presentation

A 29-year-old male patient with no comorbidities presented with abdominal pain, fever, and yellow discoloration of the eyes for 10 days. On admission, the patient was malnourished with icterus, abdominal distension, and shifting dullness. On examination, BP was 70/40 mm Hg and pulse rate 100/min. Ultrasound abdomen showed hepatosplenomegaly with ascites. He was not on any previous treatment and his covid RT-PCR was negative. Blood culture showed growth of *Salmonella typhimurium*.

The patient was treated with antifungals, ciprofloxacin, and amikacin for typhoid. He developed melaena two weeks later and was not responding to conservative management. Hence, exploratory laparotomy with ileocolic resection and anastomosis was done. Post-op blood culture showed no growth. Gross examination of the right hemicolectomy specimen showed a necrotic polypoid mass protruding into the lumen of the thinned-out terminal ileum with extensive mucosal congestion and ulceration.

**Figure 1:**
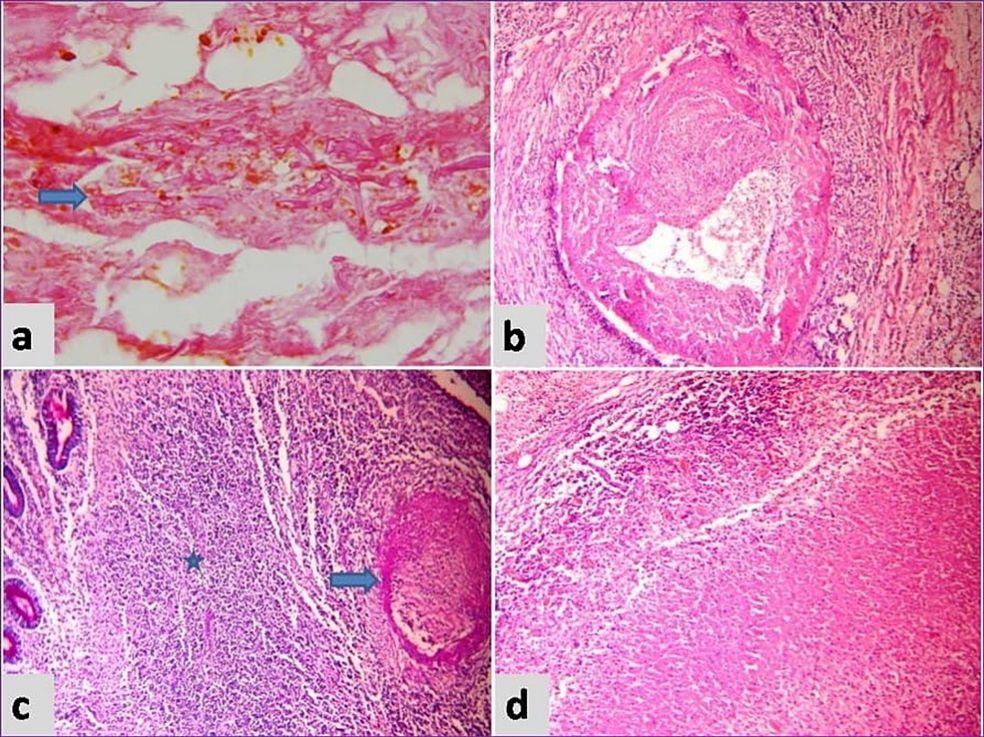
a) Necrotic tissue with fungal hyphae, H&E x400 b)Thrombosed blood vessel, H&E x200 c) Peyer's patch (star) and thrombosed vessel (arrow), PAS x100 d) Necrotic lymph node, H&E x100

**Figure 2:**
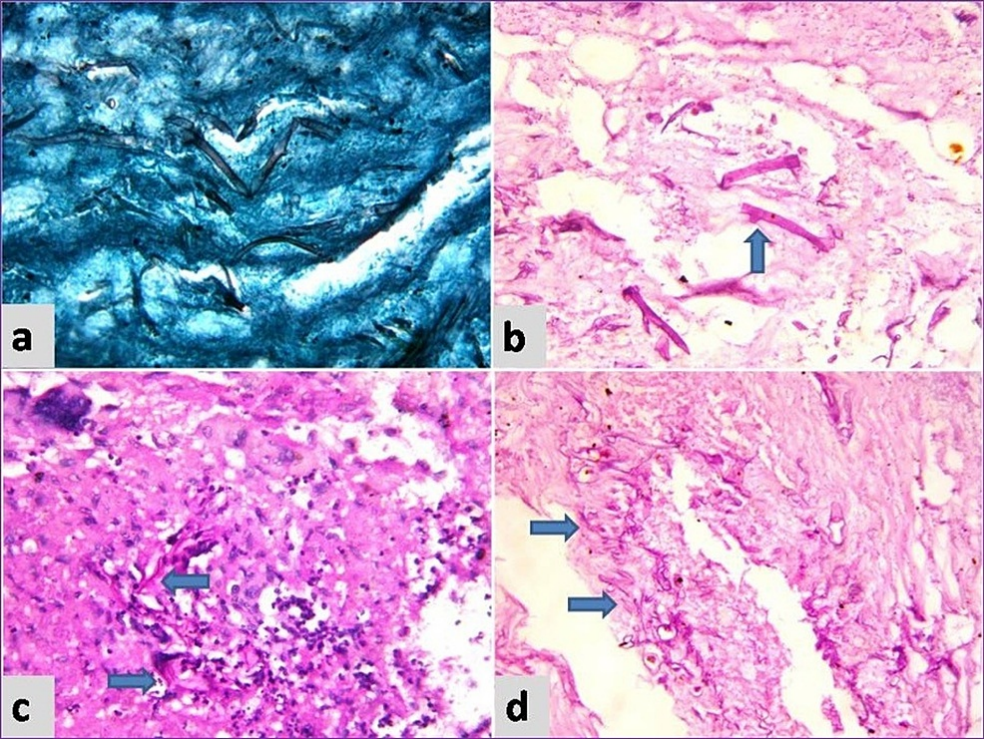
a) GMS stain showing fungal hyphae, x400 b) PAS stain showing right-angled branching (arrow), x400 c) Fungal hyphae within necrotic lymph node, PAS x400 d) fungal hyphae invading vessel wall, PAS x400

Microscopy exhibited transmural necrosis of the ileal wall with acute inflammatory exudate. The intestinal wall, Peyer's patches, and mesenteric lymph nodes showed necrosis (Figure 1). Many aseptate broad based irregular hyphae were seen in the intestinal wall morphologically suggestive of *Mucorales* (Figure 2). Thrombosis of many small and large vessels was also seen occluding the lumen (Figure 1b).

The patient was discharged in satisfactory condition on posaconazole and antibiotics.

## Discussion

Mucormycosis is a fatal infection caused by fungi belonging to the subphylum Mucoromycotina and order Mucorales [5]. GIM (Gastro-intestinal Mucormycosis) accounts for only 7% of all cases, but the mortality rate can be as high as 85%. The most common site of GI mucormycosis is the stomach followed by the colon and ileum [3,6].

Typical risk factors of mucormycosis are diabetes, defects in host phagocytes resulting in the immunocompromised state, corticosteroid use, immunosuppression for organ or stem cell transplantation, and increased levels of serum iron as a result of acidosis or administration of deferoxamine [2,3,7]. GIM has also been reported in the literature in individuals without the typical risk factors of uncontrolled diabetes mellitus or immunosuppression [2,3,8,9,10]. This is called healthcare-associated gastrointestinal mucormycosis occurring in immunocompetent adults who are admitted to the intensive care unit or after prolonged hospitalization and major surgery [11].

GI mucormycosis with concurrent typhoid fever has been very rarely reported in the literature [4]. Pathophysiology of GI mucormycosis in typhoid includes malnutrition, impaired mucosal integrity following *S. typhimurium* enteritis facilitates hyphal invasion into the intestinal wall and impaired phagocytic function of macrophages in typhoid infection further facilitates the growth of fungi [12]. Angioinvasion by the fungi, resulting in thrombosis of vessels and local ischemic necrosis, provides the nidus for hematogenous dissemination [13].

Diagnosis of GIM can often be delayed because of non-specific presentation. The most common symptoms are non-specific abdominal pain and distention associated with nausea and vomiting. Fever and haematochezia can be present. The diagnosis is usually during surgery or endoscopy by biopsy of the suspected area [3]. Successful management requires timely diagnosis, reversal of predisposing risk factors, prompt antifungal therapy, and early surgical debridement [7]. Treatment options include amphotericin B, triazole, Posaconazole [14].

## Conclusions

Association of GI mucormycosis with enteric fever is very rare. It assumes importance in developing countries where *Salmonella* infections are very common. The exact mechanism of their association is not well understood but can be attributed to disturbed host immunity in typhoid infections or due to prolonged hospital admission in immunocompetent persons.

Patients not improving on conservative management of typhoid and presenting with melaena warrant exploration. It is important to have a high index of suspicion in these patients so that early identification of such a life-threatening infection can be made.
